# Diagnostic value of the kappa free light chain index in multiple sclerosis under the 2024 CSF positivity criterion: a multicentre study in oligoclonal band-negative patients

**DOI:** 10.3389/fimmu.2026.1848491

**Published:** 2026-09-03

**Authors:** Diana Ferraro, Alessia Fiore, Elena Di Sabatino, Paola Cavalla, Antonio Gallo, Massimiliano Calabrese, Marta Radelli, Valentina Torri Clerici, Federica Pinardi, Simona Malucchi, Emilio Portaccio, Paolo Ragonese, Carolina Gabri Nicoletti, Cinzia Cordioli, Clara Guaschino, Marinella Clerico, Viviana Nociti, Eleonora Cocco, Virginia Gallina, Claudio Gasperini, Claudio Solaro, Massimiliano Di Filippo

**Affiliations:** 1 Neurology Unit, Department of Neurosciences, Ospedale Civile di Baggiovara, Azienda Ospedaliero-Universitaria di Modena, Modena, Italy; 2 Section of Neurology, Department of Medicine and Surgery, University of Perugia, Perugia, Italy; 3 Department of Neuroscience, University of Turin; Multiple Sclerosis Centre, 1st Division of Neurology, AOU City of Health and Science University Hospital, Turin, Italy; 4 Department of Advanced Medical and Surgical Sciences (DAMSS), University of Campania “Luigi Vanvitelli”, Naples, Italy; 5 Department of Neuroscience, Biomedicine and Movement Sciences, University of Verona, Verona, Italy; 6 Department of Neurology, Papa Giovanni Paolo XXIII Hospital, Bergamo, Italy; 7 Department of Neuroimmunology and Neuromuscolar Diseases, Neurological Institute C. Besta IRCCS Foundation, Milan, Italy; 8 IRCCS Institute of Neurological Sciences of Bologna, UOSI Multiple Sclerosis Rehabilitation Unit, Bologna, Italy; 9 Department of Neurology and Regional Multiple Sclerosis Reference Centres (CRESM), University Hospital San Luigi Gonzaga, Orbassano, Italy; 10 Department of Neuroscience, Psychology, Drug Research and Child Health (NEUROFARBA), University of Florence, Florence, Italy; 11 Department of Biomedicine, Neurosciences and Advanced Diagnostics, University of Palermo, Palermo, Italy; 12 Departmental School of Medicine and Health Sciences, Saint Camillus International University of Health and Medical Sciences, Rome, Italy; 13 Multiple Sclerosis Clinical and Research Unit, Department of Systems Medicine, University of Rome Tor Vergata, Rome, Italy; 14 Multiple Sclerosis Centres, ASST Spedali Civili di Brescia, Brescia, Italy; 15 Neuroimmunology Unit, Multiple Sclerosis Centre, Sant’Antonio Abate Hospital Gallarate, ASST Valle Olona, Gallarate, VA, Italy; 16 Department of Clinical and Biological Sciences, University of Turin, Orbassano, Italy; 17 Fondazione Policlinico Universitario ‘Agostino Gemelli’ IRCCS, Rome, Italy; 18 Multiple Sclerosis Research Centres ‘Prof.ssa Anna Paola Batocchi’, Catholic University of the Sacred Heart, Rome, Italy; 19 Multiple Sclerosis Centre, Binaghi Hospital, ASL Cagliari, Cagliari, Italy; 20 Department of Medical Science and Public Health, University of Cagliari, Cagliari, Italy; 21 Department of Neurosciences, San Camillo Forlanini Hospital, Rome, Italy; 22 Neurology Unit, Galliera Hospital Genova, Genoa, Italy

**Keywords:** cerebrospinal fluid, diagnostic criteria, multiple sclerosis, oligoclonal bands, κ-FLC index

## Abstract

**Introduction:**

The kappa free light chain index (κ-FLC) has been established as an alternative to oligoclonal bands (OCB) for defining cerebrospinal fluid (CSF) positivity, which can contribute to a multiple sclerosis (MS) diagnosis by the 2024 revised MS diagnostic criteria. This study aimed to evaluate the contribution of the κ-FLC index to the diagnosis of MS, through the CSF positivity criterion, in OCB-negative patients.

**Methods:**

OCB-negative patients, diagnosed with MS at any time according to the 2017 McDonald criteria, were included in this multicentre retrospective study. CSF positivity was defined by a κ-FLC index ≥ 6.1.

**Results:**

Among 141 enrolled patients, 117(83%) patients had measurable CSF κ-FLC, with a median κ-FLC index of 7.2 (IQR: 4.5-12.7) and 61 patients (43%) had a positive (≥ 6.1) κ-FLC index. A MS diagnosis was met at the time of the lumbar puncture in 102 patients, through the fulfilment of both the 2017 dissemination in space (DIS) and time (DIT) criteria. Among the remaining 39 patients, MS diagnosis was met following a relapse in 11 (28%) and based on follow-up MRI findings in 28 patients (72%). Among the 34 patients meeting DIS but not DIT criteria, 16 (47%) had a κ-FLC index ≥ 6.1. Considering the contribution of the κ-FLC index to the CSF positivity criterion, a MS diagnosis could have been anticipated in these patients by a median of 4.9 months (IQR: 1.8–14.1).

**Conclusion:**

In the context of the CSF positivity criterion of the revised 2024 MS diagnostic criteria, the κ-FLC index may contribute to an earlier MS diagnosis in OCB-negative patients fulfilling the DIS but not the DIT criteria.

## 1 Introduction

Intrathecal kappa free light chain (κ-FLC) synthesis, which can be assessed using the κ-FLC index (i.e., cerebrospinal fluid [CSF]/serum κ-FLC concentrations proportioned to CSF/serum albumin concentrations), has emerged as a more sensitive marker of intrathecal immunoglobulin (Ig) synthesis compared to IgG oligoclonal bands (OCB) in multiple sclerosis (MS) (1–4). Also, by determining the κ-FLC index, the methodological drawbacks involved in OCB analysis, such as the requirement for experienced technicians to perform and interpret the isoelectric focusing (IEF) and the relatively low inter-laboratory reproducibility, can be avoided (5, 6). The κ-FLC index is a quantitative, automated, and cost-effective measure, with an operator-independent read-out, although awareness of possible pre-analytical (e.g blood contamination and storage conditions) and biological (e.g. previous steroid/immunosuppressive treatment or renal insufficiency) confounders is warranted (7–10). Nevertheless, it is considered robust to common pre-analytical variables, and encouraging data support acceptable inter-laboratory and inter-assay concordance (11, 12), making it a practical alternative for routine clinical use (2, 13–15).

The recently published 2024 revisions of the McDonald diagnostic criteria for MS have formally included the κ-FLC index as a valid paraclinical tool, interchangeable with OCB, for defining cerebrospinal fluid (CSF) positivity and, in turn, for supporting a diagnosis of MS in both symptomatic and asymptomatic cases (16). Specifically, in individuals with typical clinical presentations and dissemination in space (DIS) involving at least two distinct central nervous system (CNS) regions, one paraclinical tool, including CSF positivity demonstrated by OCB or κ-FLC index, is sufficient to establish an MS diagnosis. DIS can be assessed by magnetic resonance imaging (MRI) or, for optic nerve involvement, by visual evoked potentials (VEP) or optical coherence tomography (OCT). Similarly, in patients with typical clinical presentations but involvement of a single topography, two paraclinical tools are required. CSF positivity, in combination with specific radiological features (i.e., presence of lesions displaying the central vein sign [CVS] or paramagnetic rim lesions [PRL]) or DIT, can contribute to an MS diagnosis. Additionally, in asymptomatic individuals with incidental detection of CNS lesions with a pattern typical of MS on MRI (i.e., radiologically isolated syndrome, RIS) or in those with non-specific neurological symptoms, a diagnosis can be established in the presence of DIS plus at least one of the following: DIT, CVS, or CSF positivity (16).

This update stems from growing and robust data showing that the κ-FLC index may identify patients with MS (pwMS) with a comparable diagnostic accuracy to OCB, and, importantly, with a concordance rate of approximately 90% (1, 13, 17, 18). Furthermore, owing to its higher sensitivity, the κ-FLC index may detect intrathecal Ig synthesis (IS) in a subset of patients who are OCB-negative at the time of the diagnostic work-up (3, 19). Thus, κ-FLC index determination, together with its larger applicability in hospitals and centres lacking specialized laboratories, can contribute to a timely MS diagnosis in a greater proportion of subjects, after careful exclusion of alternative diagnoses.

The main aim of the present study was to assess the contribution of the κ-FLC index to an MS diagnosis, through fulfilment of the CSF positivity criterion, in the framework of the 2024 diagnostic criteria in a cohort of OCB-negative pwMS.

## 2 Methods and materials

### 2.1 Patient selection

Sixteen Italian MS tertiary centres, all part of the Rising Italian Researchers in MS (RIREMS) study group, routinely employing κ-FLC index measurement in clinical practice, participated in this multicentre, retrospective, and observational study. Eligible subjects, who underwent CSF analysis during the MS diagnostic work-up, were identified within the electronic registry of each centre according to the following inclusion criteria (1): age ≥ 18 years (2); a diagnosis of MS in accordance with the 2017 revisions of the McDonald criteria and established at any time up to the last follow-up (20) (3); absence of CSF-restricted OCB (i.e., < 2 CSF-restricted OCB), including individuals displaying a “mirror pattern” or a single CSF IgG band (21) (4); availability of MRI data at the time of the diagnostic lumbar puncture. Approval number of the ethics committee of the coordinating centre (CER Umbria): 4919/25.

### 2.2 Collected information and diagnosis reclassification

Demographic, clinical, laboratory, and MRI data were anonymously collected in an electronic database. The database was locked on March 31, 2025. The following data were recorded (1): sex; (2) age; (3) date and type of onset symptoms; (4) previous steroid treatment, presence of renal insufficiency or monoclonal gammopathy; (5) presence of DIS, according to the 2017 and 2024 MS diagnostic criteria (16, 20) at the time of lumbar puncture (6); presence of dissemination in time (DIT) (20) at the time of lumbar puncture (7); MS diagnosis established either at the time of the lumbar puncture or subsequently, specifying, in the latter case, the date of diagnosis and the way of conversion (defined clinically or radiologically, by the occurrence of a subsequent relapse or of a new MRI T2 or gadolinium-enhancing lesion) (8); IEF pattern (9); IgG index values, when available (10); CSF/serum κ-FLC and CSF/serum albumin values, when reported; and (11) κ-FLC index values.

For MS reclassification according to the 2024 McDonald criteria, we focused on individuals who fulfilled the 2017 DIS (presence of at least one T2-hyperintense lesion in at least two among periventricular, juxtacortical or cortical, infratentorial, and spinal cord regions) but not DIT criteria at the time of the diagnostic lumbar puncture (baseline). A κ-FLC index cut-off of 6.1 was applied to define CSF positivity (1, 22).

### 2.3 Laboratory methods

OCB were detected and interpreted locally at each participating centre as part of routine clinical practice using IEF on paired CSF and serum samples followed by IgG-specific immunodetection. Eleven centres used the semi-automated Hydrasys^®^ platform with the Hydragel CSF Isofocusing assay (Sebia, Lisses, France), which combines agarose gel IEF with IgG-specific immunofixation. Five centres employed validated in-house IEF protocols with IgG immunoblotting or immunofixation. OCB interpretation was performed locally by experienced laboratory personnel according to standard operating procedures consistent with international and national consensus recommendations (23).

CSF and serum κ-FLC concentrations were determined locally at each participating centre as part of routine clinical practice, following internal laboratory validation procedures. Measurements were performed using either a turbidimetric assay on the Optilite^®^ Analyzer (The Binding Site Group, Birmingham, UK) with the Freelite^®^ Human Kappa Free Kit (9 centres), or a nephelometric assay on the BN II System (6 centres) or Atellica^®^ NEPH 630 System (1 centre) (Siemens Healthineers, Erlangen, Germany) using the N Latex FLC Kappa Kit. All measurements were performed according to the manufacturers’ instructions, using the corresponding proprietary calibration systems.

The lower limit of detection (LLOD) for CSF κ-FLC was 0.27–0.30 mg/L for the turbidimetric assay and 0.03 mg/L for the nephelometric assay. No κ-FLC index was calculated for samples with CSF κ-FLC concentrations below the respective assay LLOD. To assess the potential impact of unmeasurable CSF κ-FLC values, a sensitivity analysis was performed by substituting the assay-specific LLOD for the CSF κ-FLC concentration, thereby calculating the maximum possible κ-FLC index for each patient. This analysis was used to estimate the proportion of patients with unmeasurable CSF κ-FLC who could potentially have been classified as κ-FLC index-positive (κ-FLC index ≥6.1).

As an exploratory analysis, on patients with complete data, comprising CSF and serum κ-FLC and albumin quotient (Qalb) values, the Reiber hyperbolic model was applied to estimate the blood-derived fraction of κ-FLC and identify evidence of IS while accounting for blood–CSF barrier function (24). The upper reference limit for blood-derived κ-FLC quotient (Qκ) ( $Q_{\kappa ,lim}$) was calculated using the hyperbolic function proposed by Reiber (24):


$$Q_{\kappa ,lim}=3.27\sqrt{Q_{Alb}^{2}+33\times 10^{-6}}-8.2\times 10^{-3}$$

Patients were considered to have intrathecal κ-FLC synthesis when $Q_{\kappa }>Q_{\kappa ,lim}$. The intrathecal fraction (IF) of κ-FLC was calculated as:


$$IF=\max(0,(1-\frac{Q_{\kappa ,lim}}{Q_{\kappa }})\times 100)$$

Patients were then further classified according to a Reiber IF threshold of 54.6%, which showed the best diagnostic accuracy compared to other explored κ-FLC metrics in separating MS from non-MS groups in a recent large study (25).

### 2.4 Statistical analysis

Continuous variables were summarized as mean ± standard deviation (SD) or median with interquartile range (IQR). Categorical variables were presented as counts and percentages. Shapiro-Wilk test was used to assess variable distribution. Comparisons among groups were made using the Wilcoxon-Mann-Whitney or Kruskal-Wallis test, as appropriate. A Kaplan-Meier curve was used to depict time from lumbar puncture to MS diagnosis according to the 2017 criteria in patients not fulfilling the 2017 MS criteria at baseline and the log-rank test was used to compare patients with κ-FLC index values ≥ 6.1 and <6.1. Analyses were carried out using STATA 17 (StataCorp, Texas, USA).

## 3 Results

### 3.1 Characteristics of the patients

We included a total of 141 pwMS, of whom 58.2% were female, with a mean age of 43.3 years (± 13.4). At the time of the diagnostic lumbar puncture, the application of the 2017 McDonald criteria resulted in a MS diagnosis in 102/141 (72.3%) patients. Overall, 136/141 subjects (96.5%) fulfilled the 2017 DIS criterion, while DIT was met by 103/141 (73%). Only 4/141 (2.8%) did not meet either 2017 DIS or DIT criteria at baseline. Twenty-seven individuals (19.1%) had received steroid treatment prior to lumbar puncture with a median distance of 21 days (IQR: 8-37). None had renal insufficiency or monoclonal gammopathy. A detailed summary of patients’ demographic, clinical, and laboratory characteristics is provided in **Table 1**.

**Table 1:** Cohort characteristics.

Characteristic of the cohort	
Demographic features	
Number	141
Age – yrs; mean ± SD	40.7 ± 12.6
Female; n (%)	82 (58.2)
Clinical symptoms at onset; n (%)	
Optic neuritis	31 (22)
Sensory	32 (22.7)
Motor	20 (14.2)
Sensory + motor	19 (13.5)
Brainstem/cerebellar	37 (26.2)
Other	2 (1.4)
DIS and/or DIT at the time of the spinal tap; n (%)	
DIS 2017	136 (96.5)
DIS 2024	138 (97.9)
DIT	103 (73)
IEF pattern; n (%)	
Polyclonal	94 (66,5)
Single CSF band	22 (15,5)
Mirror pattern	25 (18)
IgG index	
Available; n (%)	135 (95,7)
Value; median (IQR)	0.5 (0.5-0.6)
κFLC	
CSF κ-FLC	
Reported; n (%)	128 (90.8)
Value; median (IQR)	0.5 (0.2-0.8)
Serum κ-FLC	
Reported; n (%)	128 (90.8)
Value; median (IQR)	14.2 (10,1-18,5)
κ-FLC index	
Value; median (IQR)	7.2 (4.5-12.7)
Steroid treatment before LP	
Yes; n (%)	27 (19.1)
Distance steroid treatment-LP - days; median (IQR)	21 (8-37.3)

Data are expressed as number (percentage), mean ± standard deviation or median and interquartile range.

Yrs, years; SD, standard deviation; DIS, dissemination in space; DIT, dissemination in time; IEF, isoelectric focusing; CSF, cerebrospinal fluid; κ-FLC, kappa free light chains; IQR, interquartile range; LP, lumbar puncture.

### 3.2 κ-FLC index distribution

Within the cohort of patients with measurable CSF κ-FLC (117/141 – 54/75 measured with the nephelometric platforms and 63/66 measured with the turbidimetric platform), median κ-FLC index was 7.2 (IQR: 4.5-12.7). A κ-FLC index ≥ 6.1 was detected in 61 patients. Following imputation of unmeasurable CSF κ-FLC values with the LLOD, no patient reached κ-FLC index values ≥ 6.1. Consequently, we could conclude that 43.3% of all patients (61/141) had a positive κ-FLC index. There was no difference in κ-FLC index values between patients with or without prior steroid treatment (6.7; IQR: 4.9-13.7 and 7.4; IQR: 4.5-11.9, respectively) or among patients with different IEF patterns, the most frequent one being a polyclonal pattern (94 patients: 66.5%) followed by a mirror pattern (28 patients, 18%). Among the 102 individuals who already fulfilled the 2017 McDonald criteria for MS at the time of diagnostic lumbar puncture, 43 (42.2%) exhibited a κ-FLC index ≥ 6.1, with a median value of 6.7 (IQR: 4.4-11.9). Of the remaining 39 participants who did not meet the 2017 diagnostic criteria for MS at the time of CSF collection, a subsequent diagnosis of MS was established during follow-up. Specifically, 11 patients (28%) converted following the occurrence of a new clinical relapse, whereas 28 patients (72%) were diagnosed based on follow-up MRI findings, after a median interval of 5.7 months (IQR: 2.9–13.2). In this subgroup, the median κ-FLC index was 8.1 (IQR: 4.6-15.4) and it was ≥ 6.1 in 18/39 subjects (46.2%). There was no difference in κ-FLC index values or in the proportion of patients with κ-FLC index values ≥ 6.1 among patients with or without a MS diagnosis according to the 2017 McDonald criteria at the time of the lumbar puncture. Furthermore, there were no differences in the collected demographic, clinical or CSF data in patients with a positive κ-FLC index fulfilling both DIS and DIT criteria or only DIS or DIT, nor were there differences between patients with DIS and DIT at onset versus patients diagnosed through a positive κ-FLC index versus the remaining patients.

### 3.3 Relationship between κ-FLC index positivity and Reibergram classification

As an exploratory analysis, the Reibergram was applied in patients with complete CSF/serum κ-FLC and albumin data (n=97) to assess IS while accounting for blood–CSF barrier function. Overall, 84 patients (86.6%) were classified above the hyperbolic reference range (IF>0%), including 24 (28.6%) with a negative κ-FLC index (<6.1). When applying the Reiber IF threshold of 54.6%, concordance with κ-FLC index positivity increased. Of the 97 patients, 49 (50.5%) had a Reiber IF >54.6%; all of them also had a κ-FLC index ≥6.1, whereas 11 additional patients had a positive κ-FLC index (≥6.1) but did not reach the Reiber IF threshold. No patient was positive according to the Reiber IF threshold alone. **Supplementary Tables 1, 2** summarize available data on κ-FLC and Qalb and on the agreement between these alternative KFLC IS metrics and κ-FLC index positivity.

### 3.4 Contribution of κ-FLC index to MS diagnosis

**Figure 1:**
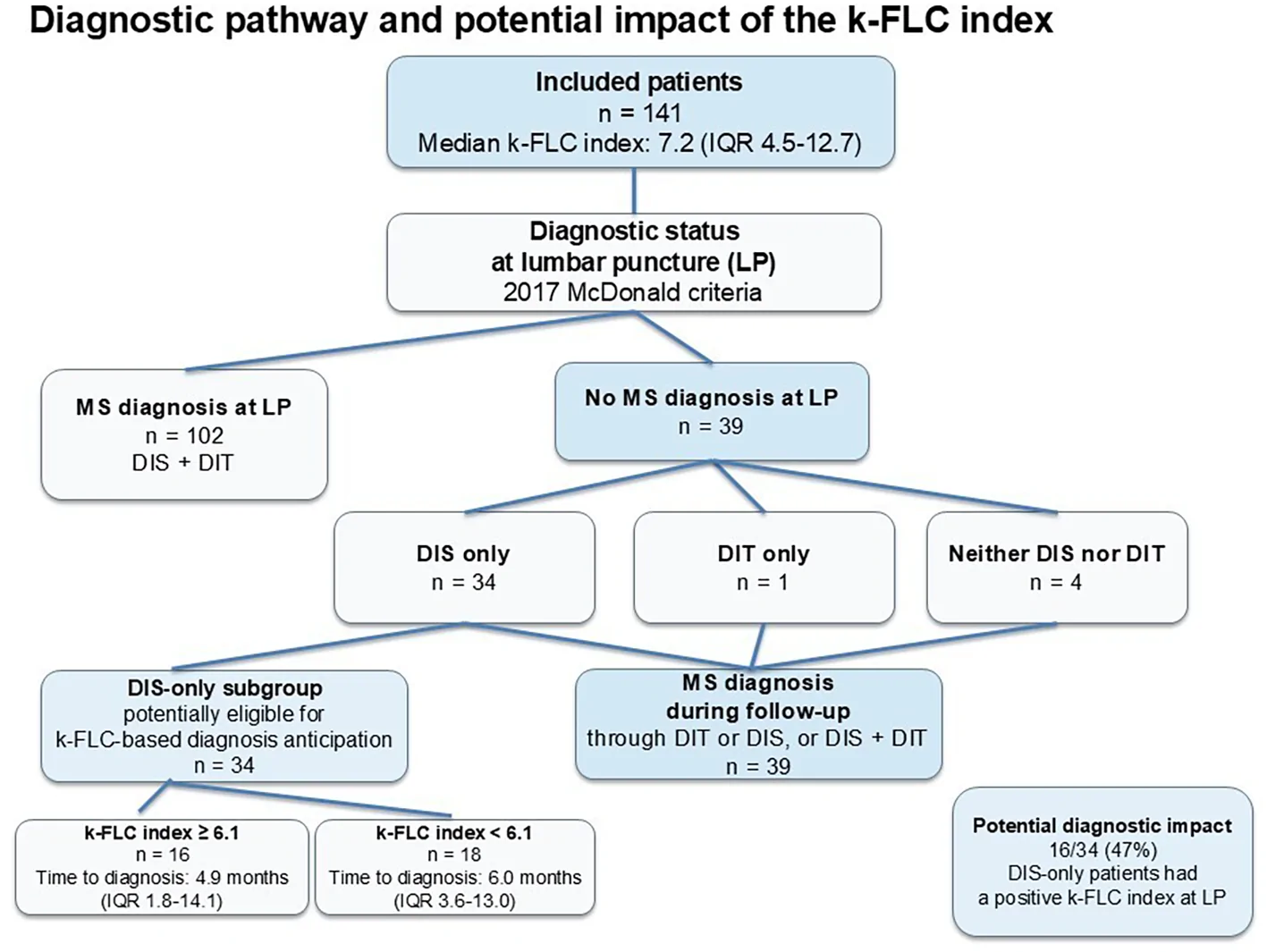
Diagnostic pathway and potential impact of kFLC index. Flowchart summarizing the diagnostic pathway and the potential clinical impact of the κ-FLC index in the study population. DIS, dissemination in space; DIT, dissemination in time; κ-FLC, kappa free light chains; IQR, interquartile range; LP, lumbar puncture; MS, multiple sclerosis.

Among the 39 patients who did not meet the 2017 diagnostic criteria at the time of the lumbar puncture, five individuals did not fulfil the 2017 DIS criterion. Considering the 34 individuals who met the 2017 DIS but not DIT criteria at the time of lumbar puncture, 16 (47.1%) had a κ-FLC index ≥6.1. Among them, a MS diagnosis was established following a clinical relapse in 7 (44%) and MRI DIT in 9 (56%) cases (**Supplementary Figure 1**). Considering the contribution of the κ-FLC index, diagnosis could have been anticipated by a median of 4.9 months (IQR: 1.8–14.1). **Figure 1** summarizes patients’ diagnostic pathway. There was no significant difference in time to MS diagnosis in patients with κ-FLC index ≥6.1 or <6.1 (Log-rank test: p=0.804) (**Supplementary Figure 2**). When the proposed 2024 DIS criterion was applied, 2 additional individuals were reclassified as fulfilling the DIS requirement (due to optic nerve MRI lesions). Neither of them exceeded the selected κ-FLC index cut-offs at the time of the lumbar puncture; however, 1 could be reclassified as MS because of the fulfilment of the DIT criterion.

## 4 Discussion

This study provides evidence supporting the contribution of the κ-FLC index in the early diagnostic setting of MS. Our findings demonstrate that κ-FLC index values ≥ 6.1 allow anticipation of an MS diagnosis, through fulfilment of the CSF positivity criterion, in nearly half of OCB-negative patients who have DIS, but not DIT, at the time of the lumbar puncture.

In the overall cohort, almost half of patients exhibited κ-FLC index values above the 6.1 cut-off despite the absence of CSF-restricted OCB. This discordance is likely multifactorial. Firstly, we cannot exclude false negative cases due to methodological limitations. OCB detection is a technically demanding procedure that requires laboratory expertise and visual interpretation of electrophoretic patterns. Despite international recommendations aimed at standardizing OCB analysis, some degree of inter-laboratory variability remains unavoidable. However, κ-FLC measurements and OCB assess related, but not identical, aspects of intrathecal B-cell activity, and discordant results between these biomarkers can be expected and have been consistently reported in previous studies (18, 25, 26). Discordant results may also in part reflect the ability of the κ-FLC index to reveal intrathecal Ig synthesis, even in the absence of IgG OCB, by reflecting the entire magnitude of humoral immunity activation in the CNS, as it is not an isotype-specific measure (5).

In the majority of our cohort, a MS diagnosis had already been established at the time of the lumbar puncture based on DIS and DIT. Among the 39 OCB-negative individuals who did not initially meet the 2017 MS diagnostic criteria, the lack of DIT was the primary reason for the absence of a baseline diagnosis, whereas almost all (34/39 individuals) had already exhibited DIS. In the latter subgroup, a κ-FLC index ≥ 6.1 could have led to an earlier MS diagnosis in approximately half of cases, with a median anticipation of 5 months. The results suggest that κ-FLC index positivity provides added diagnostic value in a proportion of OCB-negative pwMS, allowing the diagnosis to be anticipated by several months. This finding may have relevant clinical implications in terms of disease reclassification, prevention of future inflammatory activity, and ultimately preservation of neurological reserve and reduction of long-term disability. Indeed, an earlier diagnosis implies the possibility of an earlier treatment with consequent benefits in terms of long-term prognosis (27).

Our results are consistent with previous studies reporting an elevation in the κ-FLC index in a relevant subset of OCB-negative pwMS. For example, two previous studies found that a κ-FLC index ≥ 6.4 and ≥ 5.8 was present in 44% and 54% of OCB-negative patients, respectively (18, 28). Another study involving OCB-negative subjects with suspected MS reported a κ-FLC index ≥ 5.8 in about one-quarter (23/92) of cases who eventually fulfilled the 2010 MS diagnostic criteria (3). However, these studies were primarily aimed at evaluating the diagnostic predictive performance of the κ-FLC index in suspected cases, rather than its role in diagnostic anticipation. This study is the first to include a large cohort of OCB-negative pwMS, specifically evaluating the contribution of the κ-FLC index in anticipating a MS diagnosis within the context of the CSF positivity criterion of the recently published 2024 revisions of the McDonald criteria.

Notably, the mean and median κ-FLC index in our cohort were significantly lower than the values typically found in OCB-positive patients (3, 4, 29, 30). This probably reflects a circumstance, related either to the patient or to an earlier disease stage, in which intrathecal Ig synthesis is weak and a detectable IgG oligoclonal response has not (or not yet) been structured. This is in line with a high proportion of patients (15.5%) displaying possible initial signs of IgG IS through a single CSF-restricted IgG band. Indeed, this finding, is generally present in a small percentage (approximately 2%) of consecutive IEF runs (31, 32). In patients with a single CSF-restricted IgG band, κ-FLC metrics such as the κ-FLC index and the κ-FLC quotient diagrams have been shown to be informative and more sensitive, pointing to IS in a substantial proportion of cases with MS or other inflammatory neurological diseases (18, 33, 34).

As mentioned, the greater capability of the κ-FLC index (compared to OCB) in capturing an intrathecal Ig synthesis can in part be explained by the κ-FLCs being synthesized also during the assembly of other Ig classes, such as IgM and IgA, in addition to IgG. Initial data on this aspect were recently provided in a study of 69 OCB-negative patients with a kappa index ≥ 5, in which additional evidence of intrathecal Ig synthesis, as demonstrated through either the Link Index, IgM/IgA index, or IgM OCBs, was observed in 59% of cases (19). In this cohort MS was the most frequent diagnosis (27/69 patients).

The majority of patients in this study have κ-FLC index values falling in the so-called κ-FLC index “grey zone”, mostly defined by the interval between 3.5 and 20 in studies proposing diagnostic testing algorithms in which the κ-FLC is suggested as a screening tool and OCB determination is avoided in patients with κ-FLC index values falling below 3.5 (in which OCB rarely test positive) or above 20 (in which OCB rarely test negative) (26, 35–37). As with OCB, the κ-FLC index is not disease-specific. A value ≥ 6.1 does not automatically indicate MS, although higher κ-FLC levels are associated with greater diagnostic specificity (38). In this regard, the quantitative nature of the κ-FLC index can be exploited, providing added value compared to the qualitative nature of OCB assessment. At the same time, a κ-FLC index value <6.1 does not exclude a MS diagnosis, nor does it exclude other CNS demyelinating or inflammatory CNS disorders in which the κ-FLC index may more frequently fall in the “grey zone” (18, 39–41). Importantly, the κ-FLC index, especially in the “grey zone”, should be evaluated in the context of the clinical suspicion and of other CSF markers of inflammation, blood-brain barrier damage and intrathecal Ig synthesis, including OCB.

With regard to other κ-FLC metrics, application of the Reiber hyperbolic reference function, which accounts for the non-linear relationship between blood-CSF barrier function and passive protein diffusion, classified 84 of 97 MS patients (86.6%) within the IS area of the quotient diagram, compared with 60 MS patients (61.9%) classified as having a positive κ-FLC index. Although the diagnostic accuracy of the different approaches could not be assessed in the present study because no non-MS control population was included, this observation is consistent with recent studies highlighting the value of Reiber-derived κ-FLC metrics for assessing intrathecal humoral immune responses. In particular, the absence of a measurable κ-FLC intrathecal fraction (IF = 0%) has been associated with a very high negative predictive value for excluding intrathecal humoral immune activation (42, 43). Notably, an IF threshold of 54.6% showed a high concordance with a positive κ-FLC index. This finding is consistent with the recent large retrospective study by Leibowitz et al., who demonstrated that an IF threshold of 54.6% achieved a diagnostic performance comparable to κ-FLC index cut-offs of 6.1 and 8.3 for distinguishing MS from non-MS patients. Importantly, they concluded that κ-FLC metrics correcting for blood–CSF barrier function while simultaneously accounting for the magnitude of IS performed better performed better than the dichotomous approach based on whether the κ-FLC quotient (Qκ) exceeds the Reiber upper reference limit (Qκlim). Nevertheless, because the κ-FLC index is simpler to calculate and easier to interpret in routine clinical practice, they recommended it as the preferred κ-FLC metric (25).

Limitations should be acknowledged. It is a retrospective study relying on registry data and it did not provide information on the specificity of the κ-FLC index in the context of MS mimics, since it only included patients with an established diagnosis of MS. Furthermore, it did not provide an overview on the impact of the κ-FLC index in permitting an MS diagnosis in the global context of the 2024 MS diagnostic criteria. In particular: a) we did not include RIS patients, since we only included patients with a MS diagnosis based on the 2017 criteria; b) the real-world context resulted in the absence of dedicated sequences for CVS and PRL detection, preventing us from evaluating whether patients with single-topography lesions (non-DIS) could have achieved a diagnosis through κ-FLC index positivity (16) (although in our study the majority of the cohort fulfilled DIS); c) we could not assess the possibility of establishing DIS using VEP or OCT, as opposed to the MRI involvement of the optic nerve, since these assessments were not systematically carried out and/or reported. Overall, this study could, therefore, only assess the contribution to a MS diagnosis through the fulfilment of the CSF positivity criterion using the κ-FLC index as a permitted, additional, paraclinical tool.

With regard to laboratory methods, the known limitations of OCB detection through IEF with its low reproducibility across laboratories and rater-dependence, should be acknowledged. Samples were not centralized and OCB detection was not carried out using the same methods across centres, reflecting the real-world setting of this study. κ-FLC measurements were also not centralized, but were carried out locally using different platforms, with differing assay detection limits. The centralized measurement of samples using nephelometry would have probably reduced the number of samples with unmeasurable CSF κ-FLC.

Despite these limitations, our results, reflecting real-world clinical settings, indicate that the κ-FLC index can play a role in a proportion of patients, in bridging the diagnostic gap between initial clinical suspicion and confirmed MS diagnosis, thus supporting its routine assessment. Its integration in the recent diagnostic framework can contribute to reducing diagnostic delays through the fulfilment of the CSF positivity criterion.

## Data Availability

The raw data supporting the conclusions of this article will be made available by the authors upon reasonable request.

## Supplementary material

Supplementary Figure 1 Time from lumbar puncture to MS diagnosis. Kaplan-Meier curve showing the time from lumbar puncture (i.e., baseline) to MS diagnosis according to the 2017 revisions of the McDonald diagnostic criteria in OCB-negative patients fulfilling DIS + CSF positivity at baseline. OCB, oligoclonal bands; DIS, dissemination in space; CSF, cerebrospinal fluid.

Supplementary Figure 2 Time from lumbar puncture to MS diagnosis according to κ-FLC index values. Kaplan-Meier curve showing the time from lumbar puncture (i.e., baseline) to MS diagnosis according to the 2017 revisions of the McDonald diagnostic criteria in OCB-negative patients fulfilling DIS with or without a positive κ-FLC index. OCB, oligoclonal bands; DIS, dissemination in space; κ-FLC, kappa free light chains.Table
