# Supplementary material for: Diagnostic value of the kappa free light chain index in multiple sclerosis under the 2024 CSF positivity criterion: a multicentre study in oligoclonal band-negative patients
Source: Front Immunol. 2026 Sep 3;17:1848491. doi: 10.3389/fimmu.2026.1848491 (PMC13583473; doi:10.3389/fimmu.2026.1848491)
Supplement: Supplementary file 1 [file Table1.docx]

**Supplementary tables**

Supplementary Table 1. Descriptive characteristics of the 97 patients included in the exploratory Reibergram analysis.

Abbreviations: CSF: cerebrospinal fluid. κ-FLC: kappa free light chains. IF: intrathecal fraction. IQR: interquartile range.

| **Variable** | **Value (n = 97)** |
| --- | --- |
| Albumin quotient (QAlb ×100), median (IQR) | **0.61 (0.40–0.78)** |
| CSF κ-FLC (mg/L), median (IQR) | **0.63 (0.40–0.98)** |
| Serum κ-FLC (mg/L), median (IQR) | **14.5 (11.3–19.2)** |
| κ-FLC quotient (Qκ ×10³), median (IQR) | **42.5 (28.4–82.2)** |
| Intrathecal fraction (IF, %), median (IQR) | **55.4 (32.3–71.5)** |
| Patients with IF >0%, n (%) | **84 (86.6)** |
| Patients with IF >54.6%, n (%) | **49 (50.5)** |

Supplementary Table 2. Cross-classification of κ-FLC index positivity and Reiber-derived intrathecal fraction (IF) categories in patients with complete paired CSF and serum Κ-FLC measurements (n = 97). Abbreviations: κ-FLC: kappa free light chains. IF: intrathecal fraction.

| **κ-FLC index** | **IF = 0%** | **IF >0–54.6%** | **IF >54.6%** | **Total** |
| --- | --- | --- | --- | --- |
| **≥6.1** | 0 (0.0%) | 11 (18.3%) | 49 (81.7%) | 60 (100%) |
|  |  |  |  |  |
| **<6.1** | 13 (35.1%) | 24 (64.9%) | 0 (0.0%) | 37 (100%) |
